# Improving sensitivity of machine learning methods for automated case identification from free-text electronic medical records

**DOI:** 10.1186/1472-6947-13-30

**Published:** 2013-03-02

**Authors:** Zubair Afzal, Martijn J Schuemie, Jan C van Blijderveen, Elif F Sen, Miriam CJM Sturkenboom, Jan A Kors

**Affiliations:** 1 Department of Medical Informatics, Erasmus Medical Center, P.O. Box 2040, Rotterdam 3000CA, Netherlands

**Keywords:** Class imbalance, Random sampling, Cost sensitive learning, Electronic health records, Improving sensitivity

## Abstract

**Background:**

Distinguishing cases from non-cases in free-text electronic medical records is an important initial step in observational epidemiological studies, but manual record validation is time-consuming and cumbersome. We compared different approaches to develop an automatic case identification system with high sensitivity to assist manual annotators.

**Methods:**

We used four different machine-learning algorithms to build case identification systems for two data sets, one comprising hepatobiliary disease patients, the other acute renal failure patients. To improve the sensitivity of the systems, we varied the imbalance ratio between positive cases and negative cases using under- and over-sampling techniques, and applied cost-sensitive learning with various misclassification costs.

**Results:**

For the hepatobiliary data set, we obtained a high sensitivity of 0.95 (on a par with manual annotators, as compared to 0.91 for a baseline classifier) with specificity 0.56. For the acute renal failure data set, sensitivity increased from 0.69 to 0.89, with specificity 0.59. Performance differences between the various machine-learning algorithms were not large. Classifiers performed best when trained on data sets with imbalance ratio below 10.

**Conclusions:**

We were able to achieve high sensitivity with moderate specificity for automatic case identification on two data sets of electronic medical records. Such a high-sensitive case identification system can be used as a pre-filter to significantly reduce the burden of manual record validation.

## Background

Electronic medical records (EMRs) are nowadays not only used for supporting the care process, but are often reused in observational epidemiological studies, e.g., to investigate the association between drugs and possible adverse events [1-3]. An important initial step in these studies is case identification, i.e., the identification of patients who have the event of interest. Case identification is particularly challenging when using EMRs because data in the EMRs are not collected for this purpose [4]. Ideally, case identification is done on data that have been coded explicitly and correctly with a structured terminology such as the International Classification of Diseases version 9 (ICD-9). However, coding is often not available. For example, in the Integrated Primary Care Information (IPCI) database [5] used in this study, almost 60% of the record lines comprise only narratives and no coded information. The non-coded part contains essential information, such as patient-reported symptoms, signs, or summaries of specialists’ letters in narrative form. This information may be critical for identification of the events. The use of non-coded data (along with the coded data) in medical records has been shown to significantly improve the identification of cases [6]. However, the most commonly used method for case identification is using coded data only [7-11]. The current workflow of epidemiological case identification typically consists of two steps: 1) issuing a broad query based on the case definition to select all potential cases from the database, and 2) manually reviewing the patient data returned by the query to distinguish true positive cases from true negative cases. Manual review of the patient data is an expensive and time-consuming task, which is becoming prohibitive with the increasing size of EMR databases. Based on our recorded data, on average about 30 patients are reviewed per hour by a trained annotator. For a data set of 20,000 patients, which is an average-sized data set in our studies, almost 650 hours (~90 days) will be required. To make case identification more efficient, manual procedures should be replaced by automated procedures as much as possible. Machine learning techniques can be employed to automatically learn case definitions from an example set of free-text EMRs. It is crucial that an automatic case identification system does not miss many positive cases, i.e., it should have a high sensitivity. This is particularly important in incidence rate studies where the goal is to find the number of new cases in a population in a given time period. Any false-positive cases returned by the system would have to be filtered out manually, and thus the classifier should also have a good specificity, effectively reducing the workload considerably as compared to a completely manual approach.

There is a substantial amount of literature on identifying and extracting information from EMRs [12]. Machine-learning methods have been used for different classifications tasks based on electronic medical records such as identification of patients with various conditions [6,13-17], automatic coding [7,18,19], identifying candidates in need of therapy [20], identifying clinical entries of interest [21], and identifying smoking status [22,23]. Schuemie et al. [24] compared several machine-learning methods for identifying patients with liver disorder from free-text medical records. These methods are usually not optimized for sensitivity but for accuracy. The topic of automatic case identification with high sensitivity has not yet been addressed.

Typically, the proportion of positive and negative cases in a data set is not equal (usually there are many more negative cases than positive cases). This imbalance affects the learning process [25]. We use two approaches to deal with the imbalance problem: sampling methods and cost-sensitive learning. Sampling methods change the number of positive or negative cases in the data set to balance their proportions improving classifiers accuracy. This is achieved by removing the majority class examples, known as under-sampling, or by adding to the minority class examples, known as over-sampling. Both under and over-sampling methods have their drawbacks as well. Under-sampling can remove some important examples from the dataset whereas over-sampling can lead to overfitting [26]. Over- and under-sampling methods, with several variations, have been successfully used to deal with imbalanced data sets [27-32]. It has also been shown that a simple random sampling method can perform equally well as some of the more sophisticated methods [33]. We propose a modified random sampling strategy to boost sensitivity. Cost-sensitive learning tackles the imbalance problem by changing the misclassification costs [34-37]. Cost-sensitive learning is shown to perform better than sampling methods in some application domains [38-40].

In this article, we focus on improving the sensitivity of machine-learning methods for case identification in epidemiological studies. We do this by dealing with the balance of positive and negative cases in the data set, which in our case consists of all potential patients returned by the broad query. A highly sensitive classifier with acceptable specificity can be used as a pre-filter in the second step of the epidemiology case identification workflow to distinguish positive cases and negative cases. The experiments are done on two epidemiological data sets using four machine-learning algorithms.

## Methods

### Data sets

Data used in this study were taken from the IPCI database [5]. The IPCI database is a longitudinal collection of EMRs from Dutch general practitioners containing medical notes (symptoms, physical examination, assessments, and diagnoses), prescriptions and indications for therapy, referrals, hospitalizations, and laboratory results of more than 1 million patients throughout the Netherlands. A patient record consists of one or more entries, where each entry pertains to a patient visit or a letter from a specialist.

We used two data sets, one with hepatobiliary disease patients and one with acute renal failure patients. These data sets are very different from each other and are taken from real-life drug-safety studies in which it is important to investigate the incidence and prevalence of the outcomes in the general population. This type of studies serves as a good example for building highly sensitive automatic case identification algorithm because they require that all the cases in the population are identified. To construct the data sets, first a broad query was issued to the IPCI database. The aim of the query was to retrieve all potential cases according to the case definition. The query included any words, misspellings, or part of the words relevant to the case definition. The sensitivity of the broad query is very high but its specificity is usually low, and therefore many of the cases retrieved by the query are likely to be negative cases.

To train the machine-learning algorithms, a random sample of the entries returned by the broad query was selected. The size of the random sample may depend on the complexity of the case definition and the disease occurrence. Our experience suggests that the size of the random sample should be a minimum of 1,000 entries to get good performance. All patients pertaining to the randomly selected entries were manually labeled as either positive or negative cases. Because the broad query might have returned an entry with circumstantial evidence but have missed the entry with the actual evidence (e.g., because of textual variation in keywords), the entire medical record (all entries) of the patients in the random sample was considered to decide on a label, not only the entry returned by the broad query. A patient was labeled as a positive case if evidence for the event was found in any of the patient’s entries. The patient was labeled as a negative case if there was no proof of the event in any of the patient’s entries.

Each random sample was manually labeled by one medical doctor. These labels are used as a gold standard. To verify the quality of the labels and to calculate inter-observer agreement, another medical doctor then labeled a small random set (n=100) from each random sample. We used Cohen’s Kappa to calculate the agreement between both annotators [41].

Hepatobiliary disease was defined as either gall stones (with or without surgery), cholecystitis, hepatotoxicity, or general hepatological cases such as hepatitis or liver cirrhosis. The broad query (see the Appendix for the query definition) retrieved 53,385 entries, of which 1,000 were randomly selected for manual labeling. These 1,000 entries pertained to 973 unique patients, of whom 656 were labeled as positive cases of hepatobiliary disease and 317 were labeled as negative cases.

Acute renal failure was defined as a diagnosis of (sub)acute kidney failure/injury/insufficiency by a specialist and hospitalization, or renal replacement therapy followed by acute onset of sepsis, operation, shock, reanimation, tumorlysissyndrome, or rhabdomyolysis. The broad query for acute renal failure patients (see Appendix) retrieved 9,986 entries, pertaining to 3,988 patients who were all manually labeled. Only 237 patients were labeled as positive cases of acute renal failure and 3,751 patients were labeled as negative cases. Of these latter, many had chronic renal failure (an explanation for the high number of chronic renal failure patients is provided in the Appendix along with the broad query).

The labeled set included one entry per patient. For positive cases, we selected the entry with the evidence or, if multiple such entries were available, one was randomly chosen. For negative cases, we randomly selected an entry. The selected entries will be called ‘seen entries’ from here onwards.

### Preprocessing

Since a medical record may contain differential diagnosis information it is important to distinguish between positive statements made by the physician, and negations and perhaps speculations. In order to remove negated and speculative assertions we use an assertion filter, similar to others [42]. We identify three sets of keywords:

– Speculation keywords: Words indicating a speculation by the physician (e.g. ’might’, ’probable’, or ’suspected’)

– Negation keywords: Words indicating a negation (e.g. ’no’, ’not’, or ’without’)

– Alternatives keywords: Words indicating potential alternatives (e.g. ’versus’, or ’or’)

Note that the medical records and these keywords are in Dutch. Any words appearing between negation or speculation keywords and the end of a sentence (demarked by a punctuation mark) were removed from the record. Similarly, all sentences containing an alternatives keyword were completely removed. The remaining text was converted to lower case and split into individual words.

After the removal of negation, speculation, and alternative assertions, all remaining individual words in an entry were treated as features (bag-of-words representation). The advantage of using the assertion filter and bag-of-words feature representation on Dutch EMRs is presented in [24]. Since the total number of features was still very high even after preprocessing, which makes machine learning computationally expensive and may also hamper the predictive accuracy of the classifier, we performed chi-square feature selection [43]. For each feature, we compared the feature distribution of the cases and non-cases by a chi-square test. If the test was significant, the feature was selected for further processing. A p-value of less than 0.05 was used as feature selection threshold. Feature selection was done as a preprocessing step in each of the cross-validation training folds of the data sets.

### Set expansion

Adding more cases (i.e. patients) in the data set is expensive because they have to be first manually validated and labeled. We used ‘set expansion’ as an alternative approach to expand the training and test set. Each labeled set consisted of positive and negative cases, one (seen) entry per case. The fact that each case typically has multiple entries, allowed us to expand the labeled sets. For a negative case, the annotator has extensively reviewed all of the entries in the patient record and found no convincing positive evidence. Although only one random entry (seen entry) was selected for a negative case, we can however use all other entries as additional negative examples for the machine-learning because none of them contained any convincing positive evidence. We call these additional negative examples the ‘implicit entries’. For a positive case, the annotator selected an entry containing convincing positive evidence (seen entry). For all other entries of a positive case, it is uncertain whether these entries also contain convincing positive evidence. These entries therefore cannot be used as positive examples for the machine-learning. We call these uncertain entries of positive cases the ‘unseen entries’.

#### Training and testing

To train and test our classifiers, we used 5-fold cross-validation. Cross validation was done at the patient level (subject-level cross-validation [44]), i.e., the data set was randomly divided in five equally sized subsets of cases. In five cross-validation runs, each time the entries pertaining to four subsets of cases were used as a training set and the entries of the remaining subset were used for testing. For training, we used two sets of entries: a set without set expansion (i.e., with only the seen entries) and a set with set expansion (i.e., with seen and implicit entries). For testing the classifiers, however, we used all entries of the patients in the test fold. The numbers of seen, implicit and unseen entries per data set are summarized in Table 1.

**Table 1:** Total number of subjects and corresponding entries in the hepatobiliary disease and acute renal failure data sets

	**Hepatobiliary disease**	**Acute renal failure**
Positive cases	656	237
Seen entries	656	237
Unseen entries	61,179	58,022
Negative cases	317	3,751
Seen entries	317	3,751
Implicit entries	27,276	319,204

All entries of the patients in the test fold were used to simulate a real-life situation where we do not know the labels of the entries pertaining to the patients returned by the broad query. We chose not to limit ourselves to the entries returned by the broad query as they may not always contain the entry with evidence (see above), but always included all entries available for each case in the test fold.

We used sensitivity and specificity measures to evaluate the performance of the classifiers. Sensitivity is defined as the true-positive recognition rate: number of true positives / (number of true positives + number of false negatives), whereas specificity is defined as the true-negative recognition rate: number of true negatives / (number of true negatives + number of false positives).

### Improving classifiers sensitivity

The imbalance of positive and negative examples in the training set effects the classifiers performance [23]. We used sampling and cost-sensitive learning approaches to improve the sensitivity of our classifiers by dealing with this imbalance.

#### Sampling

Given an initially imbalanced data set, our proposed random sampling strategy focuses on increasing the proportion of positive case entries in the data set. Because the standard classifiers are biased towards the majority class [45-47], this improvement will potentially help the learning algorithms to generate models that better predict the positive cases, and thus improve sensitivity. In under-sampling, we only removed entries of negative cases regardless of their being in the majority or minority. For the data set with set expansion, under-sampling was done only on the implicit entries (cf. Table 1), varying from 10% under-sampling to 100% (all implicit entries removed). Thus, each negative case was left with at least one entry (the seen entry). For the data set without set expansion, under-sampling was done on the seen entries, effectively removing negative cases from the data set.

In our random over-sampling approach, we duplicated the entries of positive cases, regardless of their being in the majority or minority. The number of entry duplications was varied between 1 and 10.

#### Cost-sensitive learning

Cost-sensitive learning methods can be categorized into two categories, direct methods and meta-learning or wrapper methods [34]. In direct cost-sensitive learning, the learning algorithm takes misclassification costs into account. These types of learning algorithms are called cost-sensitive algorithms. In meta-learning, any learning algorithm, including cost-insensitive algorithms, is made cost-sensitive without actually modifying the algorithm.

We chose to use MetaCost [48], a meta-learning approach, in its Weka implementation [49]. Given a learning algorithm and a cost matrix, MetaCost generates multiple bootstrap samples of the training data, each of which is used to train a classifier. The classifiers are then combined through a majority-voting scheme to determine the probability of each example belonging to each class. The original training examples in the data set are then relabeled based on a conditional risk function and the cost matrix [48]. The relabeled training data are then used to create a final classifier.

The cost of misclassification is often not known and there are no standard guidelines available for setting up the cost matrix. Some researchers have used the ratio of positives to negatives as the misclassification cost (20) but this has been questioned by others (21). The values in the cost matrix are also dependent on the base classifier used. Some classifiers require a small misclassification cost while others require a large misclassification cost to achieve the same result. In our experiments, we varied the misclassification costs from 1 to 1000 in 9 steps.

### Classifiers

We selected the four top-performing algorithms from a previous study [24], in which many well-known machine-learning algorithms were evaluated for the classification of EMRs in a similar experimental setting.

C4.5 [50], a well-known decision-tree learner. Weka’s implementation of C4.5 (called J48) is used in the experiments.

Support Vector Machines (SVM) [46], a commonly used algorithm that can handle large data sets. Weka’s implementation of libsvm [51] is used in the experiments. Because we had a large number of binary features, we used a linear kernel [52] and the soft margin parameter *c* was set to 4.

RIPPER [53], a decision-rule learner. RIPPER induces an ordered set of rules by combining covering with a reduced error pruning strategy. Weka’s implementation of RIPPER (called JRip) is used in the experiments.

MyC, a locally developed decision-tree learner. MyC builds a tree by iteratively splitting the data based on the chi-square test, similar to the ID3 algorithm [54]. MyC is simple and very fast.

We did an error analysis to understand why some of the positive cases were not identified by the classifiers. Errors were divided in the following four categories: evidence keywords not picked up by the algorithm, evidence keyword picked up by the algorithm but removed from the patient entry by the negation/speculation filter, different spelling variations of the evidence keywords in the learned model and in the evidence entry, and patient wrongly labeled as a positive case by the annotator.

## Results

There was a good to excellent agreement between the two annotators (kappa scores of 0.74 (95% CI 0.59-0.89) and 0.90 (95% CI 0.83-0.97) for the hepatobiliary and acute renal failure data sets, respectively). The chi-square feature selection decreased the number of features in both data sets by about a factor of 10, without affecting the performance of the classifiers but greatly reducing their training time. For example, RIPPER using MetaCost took about five days to build one classifier for the acute renal failure set, which after feature selection took less than one day.

Table 2 shows the sensitivity and specificity results of all four classifiers trained on the hepatobiliary and the acute renal failure data sets, with and without set expansion.

**Table 2:** Sensitivity and specificity results of various classifiers trained on the hepatobiliary and the acute renal failure data sets, with and without set expansion

	**Set**	**Imbalance**	**SVM**		**C4.5**		**MyC**		**RIPPER**	
**Data set**	**expansion**	**ratio**	**Sens**	**Spec**	**Sens**	**Spec**	**Sens**	**Spec**	**Sens**	**Spec**
Hepatobiliary	No	0.5	0.99	0.03	0.99	0.03	0.99	0.07	0.99	0.04
	Yes	42	0.89	0.77	0.90	0.79	0.92	0.69	0.91	0.71
Acute renal failure	No	16	0.62	0.92	0.69	0.88	0.69	0.90	0.71	0.89
	Yes	1363	0.39	0.98	-	-	0.45	0.99	0.41	0.98

C4.5 could not generate a classifier for our largest data set, acute renal failure with set expansion, because the memory requirement of this algorithm proved prohibitive.

The decision-tree and decision-rule learners performed slightly better than the SVM. The imbalance ratios (number of negative examples divided by number of positive examples) varies greatly for the baseline classifiers. The specificity of the classifiers trained on the hepatobiliary data without set expansion was very low. For our sampling and cost-sensitive experiments, we therefore focused on changing the imbalance ratio in the data with set expansion. The acute renal failure data with set expansion was very imbalanced, which resulted in classifiers with relatively low sensitivity. We therefore focused on changing the imbalance ratio in the data without set expansion.

Tables 3, 4, 5 and 6 show the results for changing the proportions of positive and negative cases in both data sets by under-sampling and over-sampling, respectively.

**Table 3:** Sensitivity and specificity of various classifiers trained on the hepatobiliary data set for difference percentages of under-sampling

**Under-sampling**	**SVM**		**MyC**		**RIPPER**		**C4.5**		**Imbalance**
**(%)**	**Sens.**	**Spec.**	**Sens.**	**Spec.**	**Sens.**	**Spec.**	**Sens.**	**Spec.**	**ratio**
0	0.89	0.77	0.92	0.68	0.91	0.71	0.90	0.79	42
10	0.89	0.76	0.93	0.65	0.91	0.75	0.90	0.80	38
20	0.89	0.75	0.93	0.63	0.91	0.73	0.91	0.79	34
30	0.89	0.76	0.94	0.61	0.93	0.72	0.90	0.78	30
40	0.89	0.73	0.93	0.60	0.92	0.69	0.91	0.77	25
50	0.90	0.70	0.93	0.58	0.92	0.71	0.91	0.76	21
60	0.90	0.71	0.94	0.56	0.92	0.72	0.92	0.73	17
70	0.91	0.67	0.95	0.55	0.91	0.72	0.92	0.70	13
80	0.92	0.64	0.94	0.49	0.92	0.73	0.92	0.68	9
90	0.94	0.52	0.91	0.60	0.93	0.67	0.93	0.59	5
100	0.99	0.12	0.99	0.07	0.99	0.03	0.99	0.14	0.5

**Table 4:** Sensitivity and specificity of various classifiers trained on the acute renal failure data set for difference percentages of under-sampling

**Under-sampling**	**SVM**		**MyC**		**RIPPER**		**C4.5**		**Imbalance**
**(%)**	**Sens.**	**Spec.**	**Sens.**	**Spec.**	**Sens.**	**Spec.**	**Sens.**	**Spec.**	**ratio**
0	0.62	0.92	0.69	0.90	0.71	0.89	0.69	0.88	16
10	0.64	0.90	0.74	0.89	0.75	0.89	0.69	0.87	14
20	0.64	0.89	0.75	0.83	0.75	0.88	0.74	0.86	13
30	0.66	0.88	0.76	0.82	0.76	0.88	0.75	0.85	11
40	0.70	0.85	0.75	0.87	0.74	0.88	0.75	0.85	9
50	0.74	0.81	0.76	0.80	0.77	0.76	0.76	0.82	8
60	0.82	0.72	0.77	0.81	0.84	0.68	0.83	0.82	6
70	0.83	0.67	0.83	0.70	0.83	0.61	0.86	0.77	5
80	0.86	0.56	0.89	0.49	0.90	0.44	0.90	0.45	3
90	0.92	0.41	0.90	0.43	0.89	0.43	0.92	0.39	2

**Table 5:** Sensitivity and specificity of various classifiers trained on the hepatobiliary data set for difference percentages of over-sampling

**Over-sampling**	**SVM**		**MyC**		**RIPPER**		**C4.5**		**Imbalance**
**(%)**	**Sens.**	**Spec.**	**Sens.**	**Spec.**	**Sens.**	**Spec.**	**Sens.**	**Spec.**	**ratio**
0	0.89	0.77	0.92	0.68	0.91	0.71	0.90	0.79	42
100	0.90	0.72	0.96	0.52	0.94	0.64	0.93	0.73	21
200	0.90	0.70	0.96	0.47	0.96	0.56	0.94	0.67	14
300	0.91	0.70	0.97	0.44	0.96	0.54	0.95	0.65	11
400	0.91	0.71	0.98	0.45	0.97	0.50	0.95	0.63	8
500	0.92	0.69	0.98	0.43	0.97	0.48	0.95	0.62	7
600	0.92	0.68	0.97	0.35	0.96	0.47	0.95	0.61	6
700	0.92	0.67	0.98	0.34	0.97	0.47	0.95	0.60	5
800	0.92	0.65	0.97	0.34	0.97	0.47	0.95	0.61	5
900	0.93	0.65	0.97	0.34	0.97	0.45	0.95	0.59	4
1000	0.93	0.64	0.97	0.35	0.96	0.44	0.95	0.59	4

**Table 6:** Sensitivity and specificity of various classifiers trained on the acute renal failure data set for difference percentages of over-sampling

**Over-sampling**	**SVM**		**MyC**		**RIPPER**		**C4.5**		**Imbalance**
**(%)**	**Sens.**	**Spec.**	**Sens.**	**Spec.**	**Sens.**	**Spec.**	**Sens.**	**Spec.**	**ratio**
0	0.62	0.92	0.69	0.90	0.75	0.89	0.69	0.88	16
100	0.66	0.86	0.78	0.80	0.81	0.76	0.74	0.75	8
200	0.71	0.81	0.84	0.71	0.84	0.65	0.77	0.67	5
300	0.74	0.77	0.89	0.59	0.88	0.65	0.80	0.65	4
400	0.76	0.73	0.89	0.51	0.86	0.64	0.81	0.61	3
500	0.77	0.69	0.89	0.48	0.84	0.64	0.82	0.60	3
600	0.78	0.66	0.91	0.48	0.89	0.59	0.82	0.60	2
700	0.82	0.60	0.92	0.43	0.89	0.54	0.82	0.60	2
800	0.82	0.57	0.94	0.37	0.86	0.60	0.82	0.61	2
900	0.83	0.55	0.93	0.36	0.89	0.53	0.83	0.61	2
1000	0.84	0.54	0.95	0.36	0.88	0.54	0.83	0.61	1

All algorithms showed consistent behavior during the under-sampling experiments. The sensitivity increased and specificity decreased as we decrease the number of negative case entries from the data set.

Almost a similar pattern is observed during the over-sampling experiments where sensitivity gradually increased and specificity decreased as we increase the number of positive case entries in the data set. MyC showed slightly more improvement in the sensitivity as compared to other algorithms but then also lower specificity.

The results for cost-sensitive learning with MetaCost using varying misclassification costs are shown in Tables 7 and 8.

**Table 7:** Sensitivity and specificity of various classifiers trained on the hepatobiliary data set for difference cost values of cost-sensitive learning

	**SVM**		**MyC**		**RIPPER**		**C4.5**	
**Cost**	**Sens.**	**Spec.**	**Sens.**	**Spec.**	**Sens.**	**Spec.**	**Sens.**	**Spec.**
1	0.86	0.78	0.90	0.68	0.93	0.67	0.89	0.71
10	0.87	0.78	0.95	0.54	0.93	0.68	0.92	0.69
25	0.87	0.79	0.96	0.47	0.93	0.67	0.92	0.69
50	0.87	0.79	0.96	0.47	0.93	0.67	0.91	0.66
100	0.87	0.79	0.96	0.47	0.93	0.67	0.92	0.66
200	0.87	0.79	0.96	0.47	0.93	0.67	0.92	0.66
400	0.87	0.79	1.00	0.09	0.97	0.24	0.99	0.12
800	0.87	0.79	1.00	0.00	1.00	0.00	1.00	0.00
1000	0.87	0.79	1.00	0.00	1.00	0.00	1.00	0.00

**Table 8:** Sensitivity and specificity of various classifiers trained on the acute renal failure data set for difference cost values of cost-sensitive learning

	**SVM**		**MyC**		**RIPPER**		**C4.5**	
**Cost**	**Sens.**	**Spec.**	**Sens.**	**Spec.**	**Sens.**	**Spec.**	**Sens.**	**Spec.**
1	0.59	0.92	0.74	0.85	0.78	0.80	0.67	0.73
10	0.59	0.92	0.81	0.63	0.78	0.80	0.73	0.69
25	0.59	0.92	0.81	0.63	0.78	0.80	0.76	0.64
50	0.59	0.92	0.89	0.35	0.78	0.80	0.78	0.60
100	0.59	0.92	1.00	0.00	0.78	0.80	0.97	0.11
200	0.59	0.92	1.00	0.00	1.00	0.00	1.00	0.00
400	0.59	0.92	1.00	0.00	1.00	0.00	1.00	0.00
800	0.59	0.92	1.00	0.00	1.00	0.00	1.00	0.00
1000	0.59	0.92	1.00	0.00	1.00	0.00	1.00	0.00

Classifiers do not seem to be very sensitive to the misclassification cost so performance variations were observed at relatively high cost values.

As an example of the sensitivity that can be achieved with the sampling methods and cost-sensitive learning while maintaining a reasonable specificity, Table 9 shows the performance of the classifiers with the highest sensitivity and a specificity of at least 0.5. Our results (cf. Tables 3, 4, 5, 6, 7 and 8) show that classifiers with high specificity than 0.5 are feasible but at the expense of a lower sensitivity.

**Table 9:** Performance of the classifiers with the highest sensitivity and a specificity of at least 0.5 on the hepatobiliary disease and acute renal failure data sets

**Data set**	**Algorithm**	**Baseline**		**Under-sampling**		**Over-sampling**		**Cost-sensitive**	
		**Sens.**	**Spec.**	**Sens.**	**Spec.**	**Sens.**	**Spec.**	**Sens.**	**Spec.**
Hepatobiliary disease	SVM	0.89	0.77	**0.94**	**0.52**	0.93	0.65	0.87	0.79
	MyC	0.92	0.68	**0.95**	**0.56**	0.94	0.54	0.95	0.54
	C4.5	0.90	0.79	0.93	0.59	**0.94**	**0.56**	0.92	0.66
	RIPPER	0.90	0.71	0.93	0.72	**0.94**	**0.51**	0.93	0.67
Acute renal failure	SVM	0.62	0.92	**0.86**	**0.56**	0.84	0.54	0.59	0.92
	MyC	0.69	0.90	0.83	0.70	**0.89**	**0.51**	0.81	0.63
	C4.5	0.69	0.88	**0.86**	**0.77**	0.83	0.61	0.78	0.60
	RIPPER	0.71	0.89	0.84	0.68	**0.89**	**0.59**	0.78	0.80

**Table 10:** Error analysis of the false negatives by the MyC classifier trained on the hepatobiliary disease data set with 70% under-sampling

**Type of error**	**N (%)**
Evidence not in the model	13 (38)
Evidence removed by negation/speculation filter	12 (35)
Spelling variations	5 (15)
Labeling error	4 (12)

The performance of sampling methods and cost-sensitive learning is compared to the baseline models of both data sets.

To get an estimate of the sensitivity and specificity of manual case identification, we compared the labels of the second annotator with the gold standard labels of annotator 1. For the hepatobiliary set, sensitivity was 0.94 and specificity was 0.83, for the acute renal failure set sensitivity was 0.96 and specificity was 0.94. Our experiments (cf. Tables 3, 4, 5, 6, 7 and 8) showed that similar sensitivity performance (or even better sensitivity for the hepatobiliary set, depending on how much specificity can be compromised in a study) could be achieved using automatic classification.

We did an error analysis of the positive cases missed by the MyC algorithm using 70% under-sampling method (sensitivity 0.95) on the hepatobiliary disease data set (Table 10). About 38% of the missed positive cases were due to the evidence keywords in the entry (e.g., leverfibrose, hepatomegalie, cholestase) not being picked up by the learning algorithm. For about a third of the missed cases, the negation/speculation filter had erroneously removed the evidence in the entry. For example, in the following entry: “Ron [O] ECHO BB: cholelithiasis, schrompelnier li? X- BOZ: matig coprostase”, the evidence “cholelithiasis” was removed by the speculation filter because the sentence ended with a question mark. Spelling variations caused about 15% of the errors (e.g., “levercirrhose” instead of “levercirrose” (“liver cirrhosis”), and 12% of the missed cases turned out to be labeling errors. For example, in the following labeled entry: “Waarschijnlijk steatosis hepatitis bij status na cholecystectomie” the GP has mentioned only a probability of the disease (“waarschijnlijk”, meaning “probable”), but the patient was labeled as a positive case.

## Discussion

In this paper we demonstrated that dealing with the proportions of positive and negative cases entries in the data sets could increase the sensitivity of machine learning methods for automated case identification. We used sampling and cost-sensitive methods on two very different data sets and with four different machine-learning algorithms.

The under-sampling and over-sampling methods performed consistently well and resulted in higher sensitivity on both data sets. Although there was no clear winner between under-sampling and over-sampling methods, under-sampling performed slightly better. For the hepatobiliary set, the best sensitivity-specificity score (by selecting the highest value of sensitivity at a specificity larger than 0.5) using over-sampling was 0.94 sensitivity and 0.56 specificity with C4.5, the best score using under-sampling was 0.95 sensitivity and 0.56 specificity with MyC, and the best score using cost sensitive learning was 0.95 sensitivity and 0.54 specificity using MyC (cf. Table 9). For the acute renal failure set, the best sensitivity-specificity score using over-sampling was 0.89 sensitivity and 0.59 specificity using RIPPER, the best score using under-sampling was 0.86 sensitivity and 0.77 specificity using C4.5, and the best score using cost-sensitive learning was 0.81 sensitivity and 0.63 specificity using MyC. Overall, C4.5 and MyC appeared to perform best.

The sampling experiments demonstrated the effect of imbalance in the data sets. The question of finding an optimal or best class distribution ratio has been studied by several researchers in the past [25,55,56]. Our experiments showed that the classifiers performed better (high sensitivity with not too low specificity) when the imbalance ratio (negative cases to positive cases) was below 10 (cf. Tables 3, 4, 5 and 6). This performance improvement between the ratios was observed in both the data sets despite the fact that they were very different from each other.

Previous studies indicate that cost-sensitive learning usually performs as well as sampling methods if not better [39]. In our experiments, cost-sensitive learning performed about equally well as sampling, but it was difficult to find an optimal cost matrix. Different classifiers treat costs differently and finding an optimal cost value depends on the data set and the classifier used. Another disadvantage of cost-sensitive learning with MetaCost is the large processing time because of its bootstrapping method. For C4.5, which requires high memory and processing capacity, MetaCost did not generate classifiers for our largest data set because processing time became prohibitive.

The positive effect of set expansion for training on the hepatobiliary disease data set can be seen in Table 2. The results show that set expansion of epidemiological data sets with relatively few negative cases can boost specificity with a modest decrease in sensitivity. For example, specificity for C4.5 increased from 0.03 to 0.79 with sensitivity decreasing from 0.99 to 0.90. On this data set, the set expansion compensated for the relatively few negative examples in the data set without set expansion. The set expansion method added new entries (implicit negative case entries, cf. Table 1) with potentially useful features unlike over-sampling, where existing negative entries in the data set would be duplicated, which could lead to the problem of over-fitting. In the acute renal failure data set, negative examples were already in majority in the training model without set expansion. Set expansion further increased the imbalance, which resulted in decreased sensitivity of below 0.5 for all classifiers.

Overall, the decision tree and rule learning algorithms appear to perform slightly better than the statistical algorithms. One important advantage of tree- and rule-learning algorithms is their ability to generate models that are easily interpretable by humans. Such models can be compared with the case definitions created by human experts.

There were some study limitations. The automatic case identification system was applied on the results of the broad query to distinguish positive cases and negative cases. If cases were missed by the broad query, they will also be missed by the automatic system. In other words the sensitivity of the automatic case identification system is bound by the sensitivity of the broad query. It would be interesting to apply the automatic system on the actual EMR database and compare it with the broad query. The rate of misspellings has shown to be larger in EMRs than in other type of documents [57] but no attempts were made to handle the misspellings in the case identification system. The end of a sentence was demarked by a punctuation mark which was not optimal as later confirmed by the error analysis. Our algorithm to find negated and speculative assertions has been developed for the Dutch language and currently is not as sophisticated and comprehensive as some of the algorithms available for English, e.g., NegEx [42] or ConText [58], and ScopeFinder [59]. To deal with such issues, we need to improve our preprocessing methods. The negation algorithm can be made more informative so it can also detect double negations.

Our strategy by dealing with the imbalance ratio in a data set with and without the set expansion will result in a highly sensitive classifier. An acceptable sensitivity-specificity score will depend on the actual requirement and type of the observational study. We would like to point out that our approach is not specific to the IPCI database or the Dutch EMRs used in this study.

## Conclusions

We were able to achieve high sensitivity (on a par with the manual annotator) on both data sets using our proposed sampling and cost-sensitive methods. During a case-identification process in an epidemiological study all records returned by the broad query need to be manually validated. An automatic case-identification system with high sensitivity and reasonable specificity can be used as a pre-filter to significantly reduce the workload by reducing the amount of records that needs to be manually validated. The specificity can then be increased during the manual validation process on the reduced set. Using manual validation on the reduced set instead of the set retrieved by the broad query could save weeks of manual work in each epidemiological study.

## Appendix

The broad query used to select potential hepatobiliary disease cases. The first four words are ICPC codes. Although this query is in Dutch, the only word that is different from English is “lever”, meaning “liver”.

“D72.” OR

“D96.” OR

“D97.” OR

“D98.” OR

(“lever” AND “meta”) OR

(“hepatocell” AND “carc”) OR

(“lever” AND “tumor”) OR

“cholangiocarc” OR

“hepatitis” OR

(“lever” AND “vervet”) OR

(“steat” AND “hepat”) OR

“cholestase” OR

“cholecyst” OR

“cholelith” OR

“galst” OR

“cirrho” OR

“cirros” OR

“ciros” OR

“hepatom” OR

“hepatoslenom” OR

(“port” AND “hypert”) OR

(“lever” AND “insuff”) OR

(“lever” AND “transpl)

The broad query used to select potential acute renal failure cases. The first three words are ICPC codes.

“U05.1” OR

“U99.1” OR

“U88” OR

“oligur” OR

“tubulus” OR

“glomerulone” OR

“anuria” OR

“urem” OR

“dialyse” OR

(“rena” AND “insuf”) OR

(“rena” AND “falen”) OR

(“nier” AND “insuf”) OR

(“nier” AND “falen”) OR

(“nier” AND “trans”) OR

(“necro” AND “tubul”) OR

(“interstit” AND “nephr”) OR

(“interstit” AND “nepfr”)

The generic term “dialyse” (“dialysis”) is used for “renal replacement therapy” which is commonly associated with chronic renal failure. However “dialyse” is sometimes also used for “acute disturbance in kidney function”. The terms (“rena” AND “insuf”), (“nier” AND “insuf”), (“rena” AND “falen”), and (“nier” AND “falen”) relate to renal/kidney insufficiency/failure, a medical condition in which kidneys fail to function adequately. Inclusion of all of these terms yielded many chronic patients but exclusion of the terms would have results in missing acute renal failure patients.

## Competing interests

The authors declare that they have no competing interests.

## Authors’ contributions

ZA carried out the experiments, and drafted the manuscript. EFS and JCvB annotated the data sets. MJS, JAK, and MCJMS supervised and coordinated the project and revised the manuscript. All authors read and approved the manuscript.

## Pre-publication history

The pre-publication history for this paper can be accessed here:

http://www.biomedcentral.com/1472-6947/13/30/prepub
